# ﻿Taxonomic notes on the genus *Chaitoregma* (Hemiptera, Aphididae, Hormaphidinae), with description of a new species from China

**DOI:** 10.3897/zookeys.1218.133287

**Published:** 2024-11-14

**Authors:** Yizhe Wang, Xiaolei Huang

**Affiliations:** 1 State Key Laboratory of Ecological Pest Control for Fujian and Taiwan Crops, College of Plant Protection, Fujian Agriculture and Forestry University, Fuzhou 350002, China Fujian Agriculture and Forestry University Fuzhou China

**Keywords:** *Chaitoregma*, China, Hormaphidinae, new species, taxonomy

## Abstract

A new aphid species, *Chaitoregmakirlia***sp. nov.**, from Fujian and Guangdong, China, is described, which feeds on bamboo. The diagnostic morphological characteristics of the new species are described and illustrated. A key to apterous viviparous females of *Chaitoregma* species is provided. The COI barcode sequence of this new species is also provided. Due to its unique morphological characteristics, the diagnosis of the genus has been revised. Other species within the genus are also reviewed and discussed.

## ﻿Introduction

The aphid genus *Chaitoregma* was established by Hille Ris Lambers and Basu in 1966, with *Oregmatattakana* Takahashi, 1925 as the type species. This small genus belongs to the tribe Cerataphidini (Aphididae, Hormaphidinae). Currently, *Chaitoregma* comprises only two species and one (non-nominotypical) subspecies: *C.tattakanatattakana* (Takahashi, 1925), *C.tattakanasuishana* (Takahashi, 1929), and *C.aderuensis* (Takahashi, 1935) (Eastop and Hille Ris Lambers 1976; Remaudière and Remaudière 1997; Fang et al. 2006; Favret 2024). These species were originally discovered on Taiwan Island, China, where they feed on various bamboo species, such as *Phyllostachyspubescens*, *Yushanianiitakayamensis*, and *Bambusa* spp. (Takahashi 1925, 1929, 1931, 1935; Qiao et al. 2018).

The genus *Chaitoregma* is characterized by cylindrical frontal horns with rounded tips and nymphs that exhibit blunt frontal horns from birth. The pronotum is fused to the head; the mesonotum, metanotum, and abdominal tergites I and VIII remain free, whereas the other segments are completely fused without distinct sutures. Roundish to irregular stippled wax facets can be found everywhere on the dorsal surface of the body, but wax glands are not present in localized groups (Hille Ris Lambers and Basu 1966; Basant and Ghosh 1985; Tao 1991).

In this study, a new species, *Chaitoregmakirlia* sp. nov., is described, found on bamboo in Fujian and Guangdong, China. A key to apterous viviparous females of *Chaitoregma* species is provided. Other species within the genus are reviewed and discussed.

## ﻿Materials and methods

### ﻿Field sampling

The specimens of the new species were collected from Wuyishan Mountain, Fujian Province, China on August 2, 2016, and Mount Lianhuashan, Guangdong Province, China on July 16, 2024. During the fieldwork, photographs of live individuals were taken using a digital camera (Canon EOS 7D plus Canon EF 100 mm f/2.8L Macro IS USM Lens).

Specimens of *C.tattakanatattakana* were collected from Kunming, Yunnan Province, China on November 9, 2017.

All samples were preserved in 95% ethanol and kept at −80 °C for further morphological measurement and molecular experiments.

### ﻿Morphological description

Aphid terminology and the morphological measurements used in this paper follow Cheng and Huang (2023) (Table 1). Specimens were examined and measurements and images were taken by using Nikon SMZ18 stereomicroscope. The measurements and the micrographs of mounted specimens were performed using a computer-connected Nikon set: Nikon Eclipse Ci-L upright microscope, 16 MP digital camera with 0.55 × adapter and imaging software NIS-Elements D v. 4.60.00. The unit of measurement in this paper is millimeter (mm).

**Table 1.** Biometric data (mean, range) of *Chaitoregmakirlia* sp. nov. and *Chaitoregmatattakanatattakana*.

Part		*Chaitoregmakirlia* sp. nov. Apterous vivipara (*n* = 8)		*Chaitoregmatattakana* Apterous vivipara (*n* = 7)	
		Mean	Range	Mean	Range
Length (mm)	BL	1.164	1.012–1.372	1.322	1.209–1.395
	BW	0.690	0.624–0.789	0.734	0.645–0.852
	WA	0.198	0.187–0.210	0.236	0.230–0.241
	Ant. I	0.031	0.025–0.037	0.033	0.030–0.040
	Ant. II	0.028	0.025–0.030	0.035	0.029–0.038
	Ant. III	0.070	0.064–0.073	0.089	0.087–0.094
	Ant. III_WD	0.029	0.027–0.033	0.029	0.027–0.030
	Ant. IV	0.047	0.040–0.052	0.056	0.052–0.063
	PT	0.022	0.018–0.027	0.020	0.016–0.025
	HF	0.225	0.201–0.254	0.273	0.245–0.286
	HF_WD	0.059	0.053–0.069	0.056	0.054–0.058
	HT	0.288	0.262–0.307	0.362	0.340–0.384
	HT_WD	0.036	0.031–0.040	0.032	0.030–0.035
	2HT	0.071	0.065–0.079	0.092	0.088–0.101
	SIPH_DW	0.029	0.024–0.033	0.032	0.028–0.038
	Cauda	0.034	0.030–0.046	0.039	0.030–0.051
	Cauda_ BW	0.065	0.058–0.070	0.078	0.072–0.086
	URS	0.047	0.040–0.051	0.053	0.051–0.056
	URS_BW	0.048	0.042–0.055	0.041	0.039–0.044
	MF	0.038	0.029–0.047	0.041	0.030–0.050
	FH	0.045	0.039–0.054	0.059	0.053–0.062
	FH_BW	0.033	0.028–0.048	0.035	0.032–0.041
	Setae on dorsum head	0.059	0.050–0.066	0.090	0.079–0.112
	Setae on abd. tergites I	0.048	0.035–0.059	0.087	0.056–0.113
	Setae on abd. tergites VIII	0.060	0.042–0.072	0.081	0.062–0.096
	Setae on Ant. III	0.026	0.020–0.045	0.032	0.026–0.036
	Distance between the apex of horns	0.121	0.109–0.135	0.103	0.098–0.110
	Setae on hind tibia	0.037	0.033–0.044	0.058	0.050–0.065
No. of setae	URS	6	6–7	6	6
	Ant. I	1	1–2	2	1–2
	Ant. II	2	2–3	2	2
	Ant. III	4	3–6	4	4–5
	Ant. IV	2	1–2	1	1
	PT	4	2–5	4	3–5
	HF	15	10–19	17	24–21
	HT	22	18–28	24	21–28
	CAUDA	5	3–7	10	8–12
	AP	11	10–13	13	11–15
	GP	11	9–19	14	11–18
	GONA	10	9–13	12	10–15
	Around SIPH	5	4–6	4	3–4
	FH	8	6–12	7	6–7
	Dorsum head	18	15–20	26	25–28
	Dorsum mesonotum	11	7–12	13	11–14
	Dorsum metanotum	11	8–13	14	12–17
	Dorsum tergites I	9	7–12	15	13–16
	Dorsum tergites VIII	11	8–16	17	15–19
Ratio (times)	BL/BW	1.69	1.60–1.80	1.81	1.59–2.01
	WA/BL	0.17	0.14–0.20	0.18	0.17–0.19
	HT/BL	0.25	0.22–0.26	0.27	0.25–0.3
	HF/BL	0.19	0.18–0.21	0.21	0.18–0.23
	PT/WA	0.11	0.09–0.13	0.09	0.07–0.10
	Ant. III/WA	0.36	0.32–0.39	0.38	0.36–0.40
	PT/Ant.IV	0.48	0.39–0.68	0.37	0.27–0.48
	URS/URS_BW	0.96	0.78–1.04	1.28	1.16–1.44
	URS/2HT	0.66	0.60–0.75	0.56	0.54–0.58
	Cauda_BW/Cauda	1.88	1.52–2.16	2.10	1.53–2.87
	HF/Ant. III	3.20	2.79–3.86	3.06	2.75–3.29
	2HT/Ant. III	1.02	0.92–1.13	1.06	0.98–1.16
	URS/Ant. III	0.66	0.56–0.74	0.59	0.57–0.63

The following abbreviations have been used: BL, body length; BW, body width; WA, whole length of antenna; Ant. I, Ant. II, Ant.III, Ant. IV, for antennal segment I, II, III, IV, respectively; Ant. III_WD, the widest diameter of Ant. III; PT, processus terminalis; HF, hind femur; HF_WD, the widest diameter of HF; HT, hind tibia; HT_WD, the widest diameter of HT; 2HT, second hind tarsal segment; SIPH, siphunculus; SIPH_DW, distal width of siphunculus; Cauda_BW, basal width of cauda; URS, ultimate rostral segment; URS_BW, basal width of URS; MF, mesosternal furca; FH, frontal horns; FH_BW, basal width of frontal horns; AP, anal plate; GP, genital plate; GONA, gonapophyses.

### ﻿DNA sequencing

Whole genomic DNA was extracted from a single individual preserved in 95% ethanol using the DNeasy Blood & Tissue Kit (Qiagen, Hilden, Germany). The standard DNA barcode gene of animals, cytochrome c oxidase subunit I (5′ region of COI) was amplified with primer LepF (5′-ATTCAACCAATCATAAAGATATTGG-3′) and LepR (5′-TAAACTTCTGGATGTCCAAAAAATCA-3′) (Foottit et al. 2008). PCR amplifications were performed in a final volume of 25 µL reaction mixture containing 2 μL of template DNA, 0.5 μL of both forward and reverse primer (10 μM), 0.25 μL of Taq DNA polymerase (5 U/μL), 17.25 μL of double distilled H2O, 2.5 μL of 10 × buffer and 2 μL of dNTP. PCR thermal regime was as follows: 5 min of initial denaturation at 95 °C, 35 cycles of 20 s at 94 °C, 30 s at 50 °C (the annealing temperature) and 2 min at 72 °C, and 10 min of final extension at 72 °C. The products of PCR were visualized by electrophoresis on a 1% agarose gel and then bidirectionally sequenced at Beijing Tsingke Biotech Co., Ltd (Beijing, China). All sequences were assembled by ContigExpress (Vector NTI Suite 6.0, InforMax Inc.), and the reliability was checked by BLAST. The COI sequence was submitted to GenBank under the accession number PP910380.

The phylogenetic analysis was performed based on the sequence of the new species and 15 COI sequences downloaded from NCBI: four sequences of *C.tattakanatattakana*, two unidentified *Chaitoregma* species sequences, and seven sequences representing seven species within the tribe Cerataphidini; two sequences representing two species within the tribe Nipponaphidini were used as outgroups (Table 2).

**Table 2.** Voucher information and GenBank accession numbers of aphid samples used in molecular data analysis.

Species	Host	Locality	GenBank accession number	References
*Astegopteryxbambusae*	Bambusoideae spp.	Fujian, China	MH821551	Li et al. (2023)
*Astegopteryxstyracophila*	Zingiberaceae spp.	Hainan, China	JX489626	Chen et al. (2014)
*Ceratovacunagraminum*	Bambusoideae spp.	Fujian, China	MH821618	Li et al. (2023)
*Ceratovacunalanigera*	Bambusoideae spp.	Fujian, China	MH821646	Li et al. (2023)
*Ceratovacunakeduensis*	*Bambusaventricosa*	Fujian, China	MH821625	Li et al. (2023)
*Chaitoregma* sp.	Bambusoideae spp.	Fujian, China	MH821702	Li et al. (2023)
*Chaitoregma* sp.	Bambusoideae spp.	Fujian, China	MH821703	Li et al. (2023)
*Chaitoregmakirlia*	Bambusoideae spp.	Fujian, China	PP910380	This study
*Chaitoregmatattakanatattakana*	Bambusoideae spp.	Yunnan, China	MH821704	Li et al. (2023)
*Chaitoregmatattakanatattakana*	Bambusoideae spp.	Yunnan, China	MH821705	Li et al. (2023)
*Chaitoregmatattakanatattakana*	Bambusoideae spp.	Yunnan, China	JX489629	Chen et al. (2014)
*Chaitoregmatattakanatattakana*	Bambusoideae spp.	Guizhou, China	JN032707	Huang et al. (2012)
*Metanipponaphislithocarpicola*	*Castanopsis* spp.	Fujian, China	JX489637	Chen et al. (2014)
*Neohormaphiswuyiensis*	*Quercus* spp.	Fujian, China	JX489762	Chen et al. (2014)
*Pseudoregmapanicola*	*Cyrtococcumpatens*	Fujian, China	MH820756	Li et al. (2023)
*Pseudoregmabambucicola*	Bambusoideae spp.	Fujian, China	MH820693	Li et al. (2023)

Multiple alignment was conducted using MUSCLE (Edgar 2004). Maximum-likelihood phylogenies were inferred using MEGA X (Tamura et al. 2021) under the GTR+G+I model for 500 bootstraps. The mean genetic distances among the *Chaitoregma* species were calculated using MEGA X (Tamura et al. 2021) under Kimura’ s two-parameter (K2P) model (Kimura 1980).

### ﻿Specimen deposition

The holotype and paratypes of the new species examined here are deposited in the Insect Systematics & Diversity Lab, Fujian Agriculture and Forestry University, Fuzhou, China.

## ﻿Taxonomy

### ﻿Genus Chaitoregma

Taxon classificationAnimaliaHemipteraAphididae

Hille Ris Lambers & Basu, 1966

047BABA8-7E49-5C64-9073-154F0770775D

Chaitoregma Hille Ris Lambers & Basu, 1966: 15; Eastop and Hille Ris Lambers 1976: 143; Ghosh et al. 1977: 102; Blackman and Eastop 1984: 258; Remaudière and Remaudière 1997: 182; Qiao and Zhang 2003: 146; Aoki and Kurosu 2010: 2; Nieto Nafría et al. 2011: 171.
Chaetoregma Tao, 1991: 41. (incorrect subsequent spelling).

#### Generic diagnosis.

In apterae, body round, flat, and strongly sclerotized. Head with 1 pair of frontal horns, cylindrical with broadly rounded tips, nymph with blunt frontal horns from birth. Head plus pronotum, meso- and metanotum, and abd. tergites I and VIII mutually free, the other abdominal tergites completely fused without sutures. Body dorsum with irregularly shaped wax facets, sometimes wax plates appear in groups along the abdominal margin. Eyes with 3 facets. Antennae 4- or 5-segmented, with primary rhinaria on the terminal segment. Rostrum short and thick. Ultimate rostral segment blunt, wedge-shaped, with 3 pairs of long primary setae. Legs normal, claws normal, first tarsal chaetotaxy: 4, 3, 2. Siphunculi pore-like, not situated on hairy cones. Cauda knobbed and constricted at base. Anal plate bilobed.

#### Distribution.

China (Fujian, Guangdong, Taiwan, Yunan), India (Darjeeling).

#### Host plants.

Various species of Bambusoideae.

#### Type species.

*Oregmatattakana* Takahashi, 1925 by original designation.

### ﻿ Chaitoregma tattakana tattakana

Taxon classificationAnimaliaHemipteraAphididae

(Takahashi, 1925)

A38E55F8-5A8B-5C59-9DAD-F785BE413E00

Oregma tattakana Takahashi, 1925: 47; Takahashi 1931: 96; Tao and Tseng 1938: 218; Shinji 1941: 1115; Chu 1957: 144; Tao 1969: 57.
Chaitoregma tattakana Hille Ris Lambers & Basu, 1966: 16; Eastop and Hille Ris Lambers 1976: 143, 327; Ghosh et al. 1977: 102; Blackman and Eastop 1984: 258; Fukatsu et al. 1994: 617; Remaudière and Remaudière 1997: 182; Stern et al. 1997: 84; Fang et al. 2006: 993; Aoki and Kurosu 2010: 22; Fang et al. 2011: 160.
Chaetoregma tattakana Tao, 1991: 42.

#### Specimens examined.

• 7 apterous viviparous females, China: Yunnan (24.886°N, 102.839°E), 9 Nov. 2017, No. HL_zld20171109_2_A to G, coll. L. D. Zeng (FAFU).

### ﻿ Chaitoregma kirlia sp. nov.

Taxon classificationAnimaliaHemipteraAphididae

F8F7A382-9BF9-5295-A4A0-882E6C342B46

https://zoobank.org/3EAB9F31-3213-4EEE-8DCF-1E0732092F78

**Figure 1.**
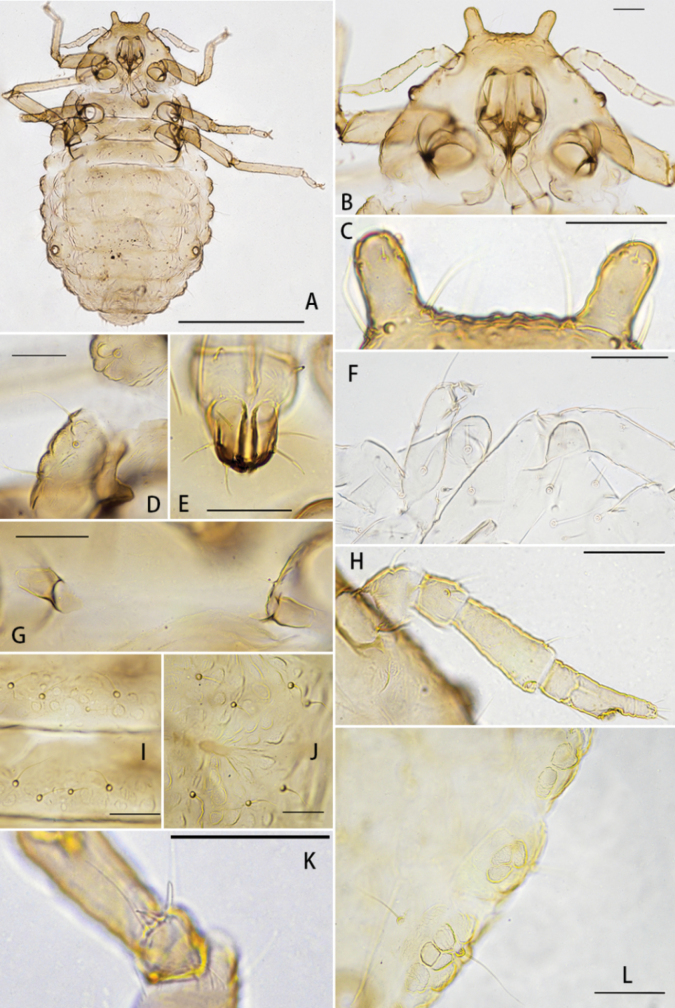
*Chaitoregmakirlia*, apterous viviparous female **A** dorsal view of body **B** head and pronotum **C** frontal horns **D** marginal wax gland plates on mesonotum **E** ultimate rostral segment **F** blunt frontal horns in embryo **G** mesosternal furca **H** antenna **I** spinal setae and wax facets on mesonotum and metanotum **J** wax facets on dorsal abdomen **K** setae on first fore tarsal joint **L** wax gland plates on marginal abdomen (**A–E, G–K** from HL_20160812_19_A; **F** from HL_20160812_19_C; **L** from HL_20160812_19_D). Scale bars: 0.5 mm (**A**); 0.05 mm (**B–L**).

**Figure 2.**
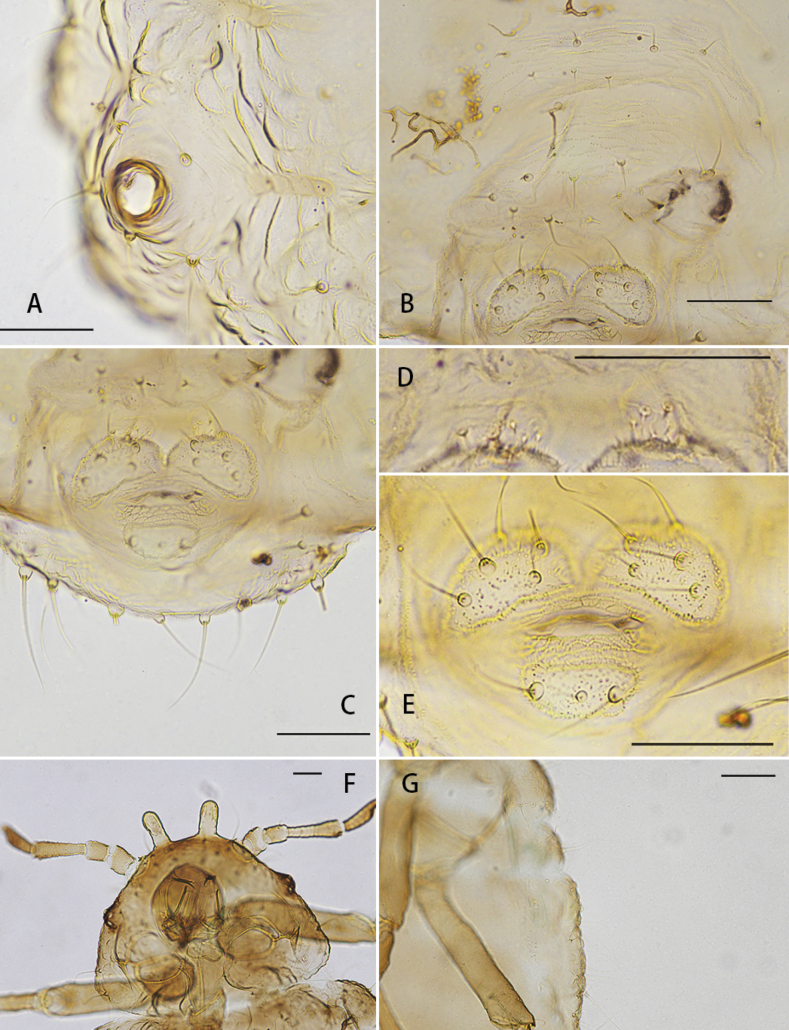
**A–E***Chaitoregmakirlia*, apterous viviparous female **A** siphunculi with 5 setae around **B** genital plate **C** abdominal tergites VIII **D** gonapophysis **E** cauda and anal plate **F, G***Chaitoregmatattakana* apterous viviparous female **F** head and pronotum **G** dorsal view of body (**A–E** from HL_20160812_19_A, **F–G** from HL_zld20171109_2_C). Scale bars: 0.05 mm (**A–G**).

**Figure 3.**
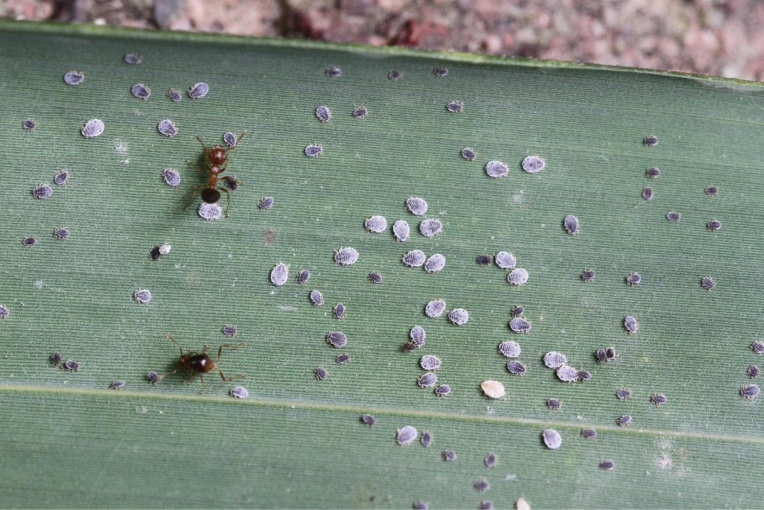
*Chaitoregmakirlia* sp. nov., colony on the underside of leaf of one undefined bamboo species, attended by an ant species, *Crematogaster* sp.

Figs 1 , 2 , 3 , Table 1

#### Etymology.

The specific epithet “kirlia” is a noun in apposition, named after Kirlia, a character from the popular Pokémon series. They both have a pair of front horns. The name was chosen to honor the graceful and elegant nature of this new species, reminiscent of the character.

#### Description.

Apterous viviparous female: body oval, dark purple in life. Body dorsum slightly covered with white wax powders, marginal areas on body with undeveloped flaky wax powders in life. For morphometric data see Table 1.

***Mounted specimens*.** Body oval and dark sclerotic (Fig. 1A), 1.62–1.82 × as long as its width, sclerotic areas evenly covered with numerous irregularly shaped wax facets, wax facets arranged radially at the intersegmental area (Fig. 1J). Head and pronotum fused (Fig. 1B), mesonotum, metanotum, abdominal segment I and VIII mutually free; abdominal segment II to VII completely fused, sutures not clearly distinct.

***Head*.** Frons with a pair of frontal horns, frontal horns cylindrical with broadly rounded tips, about 1.2–1.7 × as long as their basal width, smooth, with 6–12 short setae (Fig. 1C). Distance between the apex of the horns about 0.109–0.125 mm. Embryo with blunt frontal horns (Fig. 1F). Antennae 4-segmented, sometimes 5-segmented, about 0.15–0.19 × body length (Fig. 1H); length in proportion of segments I–IV: 25–37, 25–30, 64–73, 40–52, and 18–27. Antennal setae all fine, long with acute apices; segments I–V with 1–2, 2–3, 3–6, 1–2 setae, respectively; apical part of processus terminalis with 2–5 setae (Fig. 1F). Length of setae on segment III 0.02–0.045 mm. Segment III narrowed toward base, sensorium very small. Eyes with 3 facets in apterae. Rostrum short, reaching or nearly reaching mid-coxae; URS wedge-shaped (Fig. 1E), about 0.60–0.75 × of second joint of hind tarsi, with 3 pairs of long primary setae. Dorsal head and pronotum with 15–20 setae, 0.050–0.066 mm, fine wavy, with acute apices.

***Thorax*.** Margin of the pronotum to metanotum each with some wax facets (Fig. 1D). Dorsal setae on thorax similar to head setae. Pronotum with 2 pairs of spinal setae and 2 pairs of marginal setae; mesonotum, and metanotum each with 2 pairs of spinal, 1–2 pair of pleural and 2 pairs of marginal setae, respectively. Mesosternal furca with 2 separated arms (Fig. 1G), each arm 1.53–2.47 × as long as basal diameter of antenna segment III. Legs short, trochanters nearly fused with femora; hind tibia 0.22–0.26 × as long as body. Setae on legs fine and slightly long; setae on hind tibia 0.90–1.27 × as long as its diameter. First tarsal chaetotaxy: 4, 3, 2. The first fore tarsal joint of the legs with 2 long setae and 2 short setae (Fig. 1K), while the first hind tarsal joint with 2 long setae.

***Abdomen*.** Abdominal tergites I–VII each with 1 pair of wax gland plates on marginal sclerites, composed with irregularly shaped to rounded wax gland facets (Fig. 1L), surrounding 1 marginal seta, wax gland facets composed with 2–5 facets. Abdominal tergites I–V each with 2 pair of spinal setae, 2–4 pair of pleural and 1 pair of marginal setae; tergites VI with 1 pair of spinal, 1 pair of pleural and 1 pair of marginal setae; tergites VI with 1 pair of spinal and 1 pair of marginal setae; tergite VIII with 8–16 setae (Fig. 2C), setae on abdominal tergite VIII 2.9–3.7 × as long as basal diameter of antennal segment III. Spiracles round, open. Siphunculae pore-like, about 0.03 mm, slightly elevated, not situated on setaceous cones (Fig. 2A). Cauda knobbed and constricted at base, with about 3–7 setae (Fig. 2E). Anal plate bilobed, with 5–7 setae on each lobe (Fig. 2E). Genital plate with 4 anterior setae and 7–9 posterior setae (Fig. 2B). Gonapophyses two, each with 5–7 setae (Fig. 2D).

#### Specimens examined.

***Holotype*** • 1 apterous viviparous female, China: Fujian (Mount Wuyishan, 27.630°N, 117.394°E, alt. 234 m), 12 Aug. 2016, HL_20160812_19_A, coll. X. L. Huang and X. L. Lin (FAFU). ***Paratypes*** • 7 apterous viviparous females (HL_20160812_19_B to D on the same slide as holotype; HL_20160812_19_E to G on another slide), with the same collection data as holotype.

#### Other examined material.

• 3 apterous viviparous females on the same slide, China: Guangdong (Mount Lianhuashan, 23.067°N, 115.241°E, Alt. 905 m), 16 July 2024, WYZ_20240716_6_A to D, coll. Y. Z. Wang (FAFU).

#### Distribution.

China: Fujian (Mount Wuyishan), Guangdong (Mount Lianhuashan).

#### Host plants.

One unknown species of Bambusoideae.

#### Biology.

According to our records, *Chaitoregmakirlia* forms large colonies on the undersides of leaves of the host plant, and can be attended by ants, *Crematogaster* sp. (Fig. 3). In the wild, it has been observed that in addition to the purple individuals of this new species within the colony, there are occasionally a few yellow individuals; these are suspected to be mixed colonies with another *Chaitoregma* species, possibly *C.tattakanasuishana* (Fig. 3). The entire life cycle is unknown.

#### Taxonomic notes.

The new species resembles the type species *C.tattakana* (Takahashi, 1925), they but differ as follows: *C.kirlia* sp. nov. has distinct wax gland plates on the margin of abd. I–VI (Fig. 1L), while other species in this genus do not have distinct wax gland plates (Qiao and Zhang 2003, Fig. 2G); The new species has a greater distance between the apex of the frontal horns (0.109–0.135 mm) compared to *C.tattakanatattakana* (0.098–0.110 mm); length of the setae on the dorsum of head (0.050–0.066 mm), abd. tergites I (0.035–0.059 mm) and VIII (0.042–0.072 mm) are significantly shorter than *C.tattakanatattakana* (0.079–0.112 mm; 0.056–0.113 mm; 0.062–0.096 mm); HT 0.22–0.26 × body length (*C.tattakanatattakana*: 0.25–0.30×), PT 0.4–0.68 × Ant.IV (*C.tattakanatattakana*: 0.27–0.48×), URS 0.78–1.04 × URS_BW (*C.tattakanatattakana*: 1.16–1.43×), URS 0.60–0.75 × 2HT (*C.tattakanatattakana*: 0.54–0.58×). Number of setae on various body parts are also different (Table 1).

According to the original description, *C.kirlia* sp. nov. differs from *C.aderuensis* at least by following: HT 0.26–0.30 mm (*C.aderuensis*: 0.37 mm); WA 0.18–0.21 mm (*C.aderuensis*: 0.23 mm).

##### ﻿Molecular analyses

**Figure 4.**
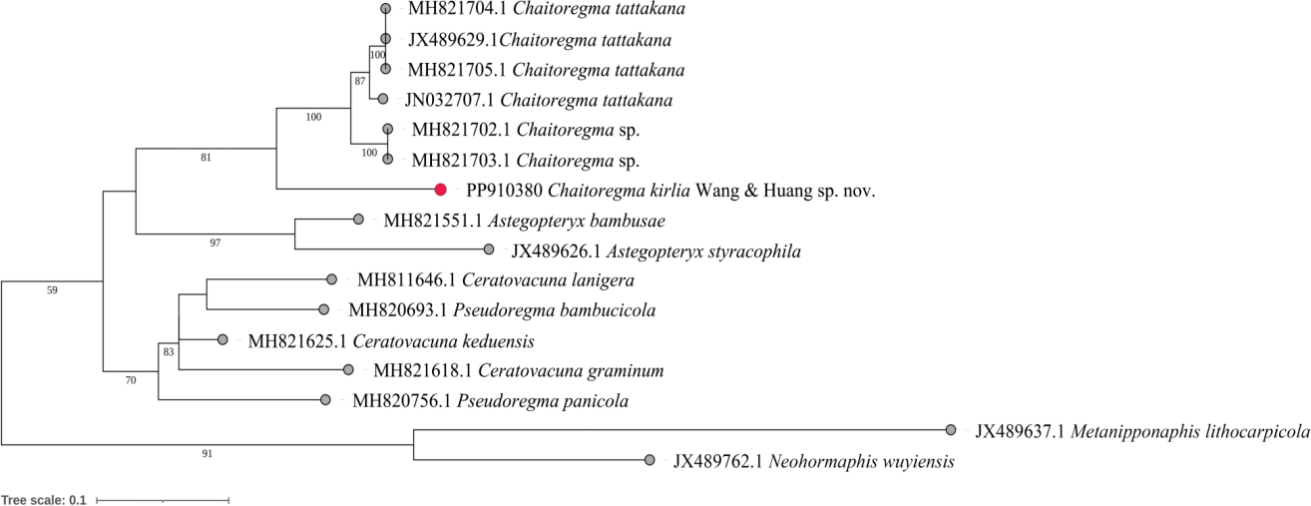
The maximum-likelihood phylogenetic tree of the samples based on COI sequences. Numbers beside main nodes are bootstrap support values (>50). Solid red circle marks the new species.

The phylogenetic results illustrate the evolutionary relationships among some species within the tribe Cerataphidini, highlighting the new species marked in red. The sequences of *C.kirlia* and *C.tattakanatattakana* cluster into two distinct clades, indicating clear genetic divergence between them (Fig. 4).

Genetic distance threshold has been used as the basis for species classification, and in aphid groups, a generally applicable threshold range is from 2% to 2.5% (Liu et al. 2013; Lee et al. 2017; Zhu et al. 2017; Li et al. 2019, 2023). The K2P distances between *C.kirlia* and other species was around 7.19–7.61% (Table 3). This significant genetic distance, exceeding the typical threshold range, supports *C.kirlia* as a distinct species.

**Table 3.** Mean genetic distances (K2P) among new species and some other species in *Chaitoregma* based on COI sequences. The percentage of genetic distances are shown in the lower left half of the matrix, and the percentage of standard errors are shown in the upper right half of the matrix.

	PP910380 *C.kirlia*	MH821705.1 *C.tattakanatattakana*	JX489629.1 *C.tattakanatattakana*	JN032707.1 *C.tattakanatattakana*	MH821702.1*C.* sp.
PP910380 *C.kirlia*		1.20	1.20	1.18	1.2
MH821705.1 *C.tattakanatattakana*	7.40		0	0.56	0.73
JX489629.1 *C.tattakanatattakana*	7.40	0		0.56	0.73
JN032707.1 *C.tattakanatattakana*	7.19	1.82	1.82		0.76
MH821702.1*C.* sp.	7.61	3.32	3.32	3.32	

## ﻿Discussion

When the genus *Chaitoregma* was established by Hille Ris Lambers and Basu (1966), they redescribed *C.tattakanatattakana* only using the samples collected from southern Himalayas. There could be some subspecific differences between these samples, which could have led to inaccuracies in their redescription.

On Blackman and Eastop’s website “Aphid on world’s plants” (Blackman and Eastop 2024), they mentioned that *C.aderuensis* was not clearly distinct from *C.tattakanatattakana* based solely on the original description. After examining the original description, we determined that the shape of the frontal horns is key in distinguishing them: the frontal horns of *C.aderuensis* are narrowed on the apical part, while the frontal horns of *C.tattakanatattakana* are broadly rounded at the apical part. This distinction is based solely on the original description, and we need more sampling in the future to confirm the relationship between these two species.

According to the original description, the subspecies *C.tattakanasuishana* can be distinguished from *C.tattakanatattakana* by its yellowish-brown body color in life, frontal horns not constricted at the base and slightly narrowed towards the apex, SIPH_DW longer, about 0.037 mm, and a slightly less sclerotic body (Takahashi 1929). These limited features indicate that *C.tattakanasuishana* should likely be considered a distinct species rather than a subspecies. In the future, we need more sampling or the opportunity to examine type specimens to clarify the relationships between these species (subspecies).

### ﻿Key to species of *Chaitoregma* (Apterous viviparous females)

**Table:** 

1	Body yellow in life	***C.tattakanasuishana***
–	Body hazy bule purple in life	**2**
2	Abdominal tergites I–VII each with 1 pair of wax gland plates on marginal sclerites, composed of irregularly-shaped to rounded wax gland facets	***C.kirlia* sp. nov.**
–	Abdominal tergites I–VII only with roundish stippled wax facets, which are not in groups	**3**
3	Head narrowed between antenna, and horns narrowed on apical part	***C.aderuensis***
–	Horns not expanded at base, not narrowed toward apex, but sometimes very slightly narrowed toward base, broadly rounded at apical part	***C.tattakanatattakana***

## Supplementary Material

XML Treatment for Chaitoregma

XML Treatment for Chaitoregma tattakana tattakana

XML Treatment for Chaitoregma kirlia

## ﻿Additional information

### Author ORCIDs

Yizhe Wang https://orcid.org/0009-0004-4703-2226

Xiaolei Huang https://orcid.org/0000-0002-6839-9922

### Data availability

All of the data that support the findings of this study are available in the main text.
